# Sonication-Induced Modification of Carbon Nanotubes: Effect on the Rheological and Thermo-Oxidative Behaviour of Polymer-Based Nanocomposites

**DOI:** 10.3390/ma11030383

**Published:** 2018-03-05

**Authors:** Rossella Arrigo, Rosalia Teresi, Cristian Gambarotti, Filippo Parisi, Giuseppe Lazzara, Nadka Tzankova Dintcheva

**Affiliations:** 1 Dipartimento di Ingegneria Civile, Ambientale, Aerospaziale, dei Materiali, Università degli Studi di Palermo, Viale delle Scienze, Ed. 6, 90128 Palermo, Italy; rosalia.teresi@unipa.it (R.T.); nadka.dintcheva@unipa.it (N.T.D.); 2 Dipartimento di Scienza Applicata e Tecnologia, Politecnico di Torino, Viale T. Michel, 5, 15121 Alessandria, Italy; 3 Dipartimento di Chimica, Materiali ed Ingegneria Chimica “G. Natta”, Politecnico di Milano, Piazza L. da Vinci 32, 20133 Milano, Italy; cristian.gambarotti@polimi.it; 4 Dipartimento di Fisica e Chimica, Università degli Studi di Palermo, Viale delle Scienze, Ed. 17, 90128 Palermo, Italy; filippo.parisi@unipa.it (F.P.); giuseppe.lazzara@unipa.it (G.L.)

**Keywords:** UHMWPE, CNTs, sonication, nanocomposites, rheology, thermo-oxidative stability

## Abstract

The aim of this work is the investigation of the effect of ultrasound treatment on the structural characteristics of carbon nanotubes (CNTs) and the consequent influence that the shortening induced by sonication exerts on the morphology, rheological behaviour and thermo-oxidative resistance of ultra-high molecular weight polyethylene (UHMWPE)-based nanocomposites. First, CNTs have been subjected to sonication for different time intervals and the performed spectroscopic and morphological analyses reveal that a dramatic decrease of the CNT’s original length occurs with increased sonication time. The reduction of the initial length of CNTs strongly affects the nanocomposite rheological behaviour, which progressively changes from solid-like to liquid-like as the CNT sonication time increases. The study of the thermo-oxidative behaviour of the investigated nanocomposites reveals that the CNT sonication has a detrimental effect on the thermo-oxidative stability of nanocomposites, especially for long exposure times. The worsening of the thermo-oxidative resistance of sonicated CNT-containing nanocomposites could be attributed to the lower thermal conductivity of low-aspect-ratio CNTs, which causes the increase of the local temperature at the polymer/nanofillers interphase, with the consequent acceleration of the degradative phenomena.

## 1. Introduction

The outstanding electrical, magnetic and mechanical properties of CNTs have motivated a flurry of interest in exploiting their potential as nanofillers in polymer-based nanocomposites [1,2]. Indeed, several studies reported in the literature show that adding CNTs to a polymer matrix causes a reinforcement of mechanical properties [3] and an enhancement of thermal and electrical conductivity [4,5]. Furthermore, the introduction of CNTs in polymeric matrices can enlarge the polymer application field, since this strategy allows the formulation of technologically relevant nanostructured materials suitable for advanced applications, such as energy storage [6], electronics [7], catalysis [8] and sensors [9].

The final properties of CNT-containing nanocomposites strongly depend on the extent of the CNT dispersion within the polymeric matrix and on the quality of the polymer/nanofiller interfacial region. Due to their high surface-to-volume ratio, CNTs tend to interact through Van der Waals forces, forming agglomerates and aggregates that compromise the obtainment of a homogeneous distribution of CNTs thorough the host matrix and, hence, a proper transfer of the CNT properties to the polymer [10]. To overcome this drawback, significant efforts have been devoted to the modification of the external surface of CNTs in order to enhance their ability to disperse in polymers or to form stable suspensions in both organic and inorganic solvents. The functionalization of CNT surfaces can be achieved by exploiting several methods, including covalent linkage [11] and physical absorption [12], and, besides ensuring improved dispersability of the CNTs in polymers, can provide them with new functionalities.

The intrinsic properties of CNTs themselves—such as surface quality [13], waviness and, above all, their aspect ratio [14]—are also of paramount importance to obtain nanostructured polymer-based materials with enhanced final properties compared to the corresponding neat matrices. Indeed, the high aspect ratio of CNTs is associated with a high surface-to-volume ratio, that helps promote the CNT superior properties to the polymer matrix [15,16]. Therefore, the improvement of the final properties of CNT-containing nanocomposites is in part attributed to the nanoparticles’ high aspect ratio, and many studies have shown enhanced physical properties of polymer nanocomposites with increasing nanofiller aspect ratio [17,18]. Kim et al. studied the effect of CNT geometry and dimensions on the thermal conductivity of polymer-based nanocomposites, showing that an effective control of this property can be achieved through the adjustment of the nanoparticle length. The dependence of the thermal conductivity of CNTs based nanocomposites on the nanofiller aspect ratio can be explained considering that a higher aspect ratio favors the formation of a percolation network, allowing the conduction of phonons over a long distance, without any transition from particle to particle [19].

The aspect ratio of CNTs also plays a fundamental role in determining the percolation threshold in polymer-based nanocomposites, that is, the critical concentration of CNTs needed to form interconnected networks of nanofillers within the polymer matrix. When the CNT concentration reaches the percolation threshold, the nanocomposite electrical conductivity (electrical percolation threshold) or storage modulus (rheological percolation threshold) suddenly increases, due to the formation of a semi-3D network of nanofillers within the host polymer matrix [20,21]. As reported in the literature, an increase of the CNT aspect ratio reduces the critical concentration for the formation of a percolated network and nanoparticles with a very high aspect ratio can form interconnected networks at very low volume fractions [22]. Cipriano et al. [23] studied the effect of the CNT aspect ratio on the rheological behavior of polystyrene-based nanocomposites, showing that CNTs with a higher aspect ratio bring about a more pronounced enhancement of both the storage modulus and viscosity compared to that of nanocomposite-containing CNTs with a lower aspect ratio. Similar results have been found by Potschke et al. in polycarbonate/CNT nanocomposites for which, at a given CNT concentration, the complex viscosity progressively increases with an increase in the CNT aspect ratio [24].

Ultrasonic treatment is widely used to disperse CNTs in solution, to functionalize CNTs or to formulate nanocomposites and nanomaterials [25,26]. The effects of sonication on CNTs derive from the shear forces coming from cavitation phenomena that are able to break the CNT aggregates formed due to the Van der Waals interactions between the CNTs themselves [27]. However, the cavitation phenomena can cause the formation of structural defects on the CNT’s surface and, eventually, their breakage, with modification of initial CNT length and aspect ratio [28].

The aim of this work is to evaluate the effect of ultrasonic treatment on the CNT aspect ratio and how this issue affects the morphology and the rheological properties of polymer-based nanocomposites, as well as their thermo-oxidative resistance. Despite qualitative and quantitative studies on reducing CNT length by sonication being present in the scientific literature, in many cases this has not been accompanied by investigating how the alteration of the CNT’s structure and aspect ratio influences the rheological behaviour and, above all, the thermo-oxidative stability of CNT-containing nanocomposites. Specifically, in this work, CNTs were subjected to a sonication treatment for different time intervals and the thus modified nanofillers were used to formulate ultra-high molecular weight polyethylene (UHMWPE)-based nanocomposites. The rheological behavior and the thermo-oxidative resistance of formulated nanocomposites have been deeply evaluated and discussed, considering the effect of the sonication time on the initial dimensions of CNTs.

## 2. Materials and Methods

### 2.1. Materials

UHMWPE is a commercial grade product purchased by Sigma-Aldrich (Saint Louis, MO, USA) in the form of a white powder. Its main properties are: average molecular weight M_w_ = 3 ÷ 6 MDa, softening point T = 136 °C (Vicat, ASTM D 1525B), melting point Tm = 138 °C, degree of crystallinity about 48.1% (estimated through Differential Scanning Calorimetry measurement considering a heat of fusion for perfectly crystalline polyethylene ∆Hf = 288.84 J/g), and density ρ = 0.94 g/mL at 25 °C. The average molecular weight was detected by means of intrinsic viscosity [η] measurements according to ASTM D4020-05 using a Lauda Pro-line PV15 viscometer. The UHMWPE was dissolved in decahydronaphtalene (reagent grade Sigma-Aldrich) at 150 °C and maintained under magnetic stirring for one hour. The viscosimetric average molecular weight, estimated from three independent measures of [η] using the Margolies equation, Mv = 5.37 × 10^4^ [η]^1.37^, was Mv ≅ 4.9 × 10^6^ g/mol.

Multiwalled carbon nanotubes, CNTs, were prepared by the typical chemical vapor deposition (CVD) protocol, using ethylene as the carbon source [29]. The purification was performed with 50% aqueous sulfuric acid, obtaining carbon nanotubes with outer diameters ranging between 14 and 20 nm, inner diameters in the range of 2 to 5 nm, length 1 ÷ 10 μm, and purity >98 wt %. After the purification process, the content of carboxylic groups was estimated to be about 0.5% by means of XPS analysis and acid/base titration.

All chemicals and reagents were used as received, without further purification.

### 2.2. Carbon Nanotubes (CNTs) Sonication

Six identical samples of 0.2 g of CNTs were placed in 50 mL one-neck round bottom flasks, then 20 mL of distilled water were added to each one. The flasks were placed in an ultrasonic bath (water volume 2.5 L, power 260 W) taking care to have the water level higher than the level of the suspension. The six reaction mixtures were sonicated at room temperature for different time intervals, particularly, 15, 30, 60, 90 and 120 min. The ultrasound treated CNTs were labeled as CNT-X, where X is the sonication time.

### 2.3. Nanocomposite Preparation

The UHMWPE powder and 1 wt % of untreated CNTs and sonicated CNTs were manually mixed at room temperature and the resulting powder was homogenized by thorough grinding in a porcelain mortar to a visually homogeneous state. Afterwards, the composite powder was kept under magnetic stirring for about 12 h, until the achievement of a homogeneous black powder.

The blends were then hot compacted at 210 °C for 5 min and under a pressure of 1500 psi to get thin films (thickness less than 100 μm) for the subsequent analyses.

The neat UHMWPE was subjected to the same procedure for comparison.

### 2.4. Characterization

To assess the surface quality of CNTs, micro-Raman spectroscopy was performed at room temperature through a Renishaw (Gloucestershire, UK) Invia Raman Microscope equipped with a 532 nm Nd:YAG laser excitation and 100 mW power. Non-confocal measurements were carried out in the range 3200–100 cm^−1^ with a spectral resolution between 0.5 and 1 cm^−1^.

Transmission electron microscopy (TEM) analyses were performed on a homogeneous dispersion of the CNTs in 2% aqueous sodium dodecyl sulfate (SDS, supplied by Sigma-Aldrich) using a Philips (Amsterdam, The Netherlands) CM 200 field emission gun microscope operating at an accelerating voltage of 200 kV, with the aim of inspecting the morphology of treated CNTs. A Gatan (Pleasanton, CA, USA) US 1000 CCD camera was used and 2048 × 2048 pixels images with 256 grey levels were recorded. For the specimen preparation, a few drops of the water solutions were deposited on 200 mesh lacey carbon-coated copper grid and air-dried for several hours before analysis.

In order to evaluate the dimensions of the ultrasound-treated CNTs, dynamic light scattering measurements on the high diluted CNT dispersions (10^−3^ *w*/*w* %) were carried out using a Zetasizer NANO-ZS (Malvern Instruments, Malvern, UK). A 5 mM sodium dodecyl sulphate (SDS) aqueous solution was used to improve the CNTs’ colloidal stability. The field-time autocorrelation functions were analysed by inverse Laplace transformation (ILT) that provide the distribution of the decay rates (Γ) for the diffusive modes. For the translational motion, the collective diffusion coefficient (D) given by D = G/q^2^, where q is the scattering vector (4πnλ − 1 sin(θ/2); with n being the water refractive index, λ the wavelength (632.8 nm), and θ the scattering angle (173°)). The distribution of the apparent hydrodynamic diameters (d_H_) was calculated by using the Stokes−Einstein equation.

The rheological behavior of formulated nanocomposites was studied through rheological tests conducted by means of a strain-controlled rotational rheometer (ARES G2 by TA Instruments, New Castle, DE, USA) in parallel plate geometry (plate diameter 25 mm). The complex viscosity (η*) was measured performing frequency sweep tests at T = 210 °C from 10^−2^ to 10^2^ rad/s at a maximum strain of 2%. As proved by preliminary strain sweep experiments, such an amplitude is low enough to be in the linear viscoelastic regime. Besides, linear stress relaxation measurements were carried out, submitting the samples to a single step strain γ_0_ = 1%, and the shear stress evolution during time σ(t) was measured to obtain the relaxation modulus G(t) = σ(t)/γ_0_. As regards the reproducibility of the obtained results, it was satisfactory (±5%).

The nanocomposite morphology was inspected using optical microscopy (Leica (Torino, Italy) Microscope) in reflection mode at a magnification of 20×. Images were acquired on the surface of the nanocomposite films.

A Fourier transform infrared spectrometer (FTIR) (Spectrum Two FTIR spectrometer, Perkin Elmer, Waltham, MA, USA) was used to record the infrared spectra. FT-IR analyses were carried out to infer the advance of the degradation phenomena on nanocomposite films. Specifically, the samples were first treated in an air oven at T = 120 °C, which is a temperature lower than the T_m_ of the polymer but high enough to accelerate the degradation processes. Then, FT-IR spectra were collected performing 16 scans between 4000 and 500 cm^−1^ on samples subjected to thermo-oxidation for different exposure times. The carbonyl index (CI) was calculated as the ratio between the carbonyl absorption area (1850–1600 cm^−1^) and the area of a reference peak at about 2019 cm^−1^.

## 3. Results and Discussion

### 3.1. Assessment of the Effect of Ultrasonic Treatment on the CNT Dimensions

**Figure 1:**
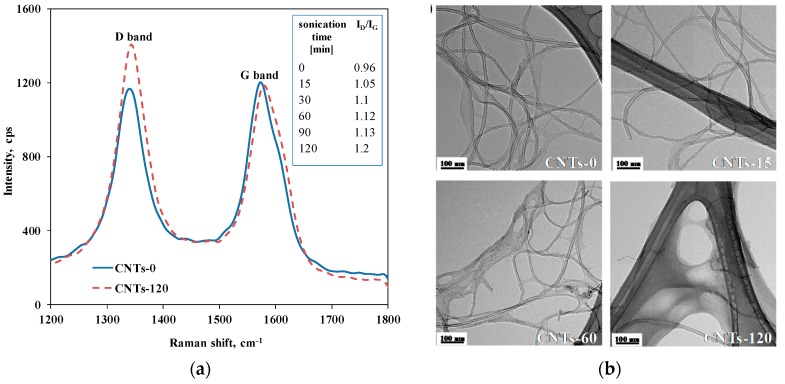
(**a**) Raman spectra of untreated CNTs and CNTs subjected to sonication for 120 min (in the inset the values of ID/IG ratio of all CNTs samples are listed); (**b**) representative TEM micrographs of investigated CNTs.

First of all, to assess the effect of sonication on the morphology and dimensions of CNTs, preliminary analyses of untreated CNTs and CNTs subjected to the sonication treatment for different time intervals were carried out. More specifically, to evaluate the effect of the treatment on the CNTs’ defect content, Raman analyses were performed. Figure 1a reports the spectra obtained for untreated CNTs and CNT-120. In the Raman spectra, two prominent peaks were detected: the D-band located at 1340 cm^−1^ and the G-band centered at about 1580 cm^−1^. The first is known as defect-induced disorder mode, and refers to the carbon atoms on the CNT surface having sp^3^ hybridization; the second is the so-called tangential mode and is related to the graphitic structure of the CNT surface [30]. In Figure 1a, each spectrum has been normalized such that the G-band intensity is constant. It is clearly visible that the intensity of the D-band is more pronounced for the CNTs subjected to the maximum sonication time than for the untreated CNTs, indicating damage to the CNT structure and the formation of lattice defects due to the sonication treatment. The unusual high intensity of the D-band for the untreated CNTs can be explained considering that the purification process can cause the modification of the CNT surface, with the anchoring of oxygen-containing groups. To qualitatively evaluate the content of structural defects, the ratio I_D_/I_G_ between the intensities of the two Raman bands has been calculated for all CNTs and the obtained values are reported in the inset in Figure 1a. Indeed, it is known from the literature that this ratio accounts for the concentration of defect site content on CNT structures [31]; specifically, a higher value of the ratio means that more defects are formed.

The I_D_/I_G_ ratio is 0.96 for the untreated CNTs progressively increased with the increasing sonication time, until it reaches a value of 1.20 for the CNT-120. This feature suggests that, due to the sonication treatment, progressive damage to the CNTs’ graphitic structure occurs, with the formation of structural defects on the CNT surface, the concentration of which rises with the increasing sonication time.

To qualitatively evaluate the morphological modifications of CNT structures associated with the defect formation on sonication treatment, a visual inspection using TEM was carried out. In Figure 1b, representative TEM micrographs of untreated CNTs and CNTs subjected to sonication for different time intervals are reported. For the sonicated CNTs, alterations of the nanoparticles’ structural integrity can be noticed, with the appearance of waviness and presence of amorphous carbon on the nanoparticle surface, while untreated CNTs show a well-ordered graphitic structure.

Defect formation on CNTs induced by the sonication treatment can lead to nanoparticle scission. To verify the effect of the sonication on the CNTs’ length, the distribution of the hydrodynamic diameter of the untreated and sonicated CNTs was evaluated by dynamic light scattering (DLS) measurements. The average apparent hydrodynamic diameters are related to the characteristic lengths of the anisotropic particles, as well as to the shell hydration and the aggregation phenomena. It is known from the literature that the apparent hydrodynamic diameter for anisotropic particles is a direct measure of the translational diffusion behaviour of the scattering objects and it can be correlated to the CNTs’ actual characteristic sizes [32,33,34]. In particular, for a cylinder, the value of the hydrodynamic diameter can be calculated by considering that [35]

(1) $$D_{h}=\frac{2L}{2s-0.19-8.24/s+12/s^{2}}$$

where L is the cylinder length, and s = ln(L/r) where r is the radius of the cylinder.

**Figure 2:**
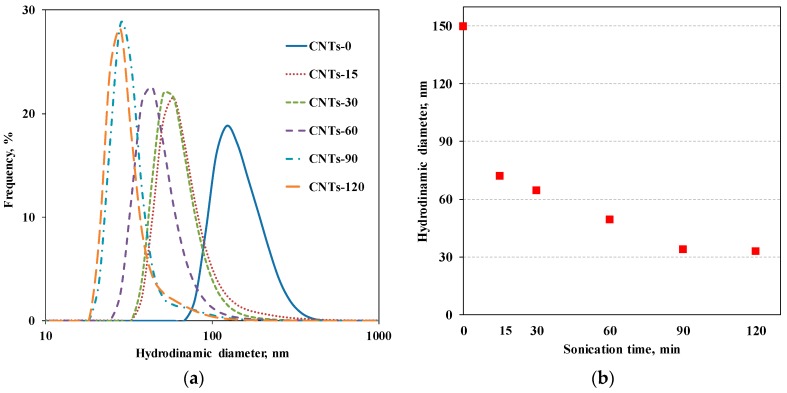
(**a**) Distribution functions of apparent hydrodynamic diameters for untreated and sonicated CNTs and (**b**) apparent hydrodynamic diameter of CNTs as a function of sonication time.

In Figure 2a, the obtained distribution functions of the average hydrodynamic diameters are reported, for untreated CNTs and for CNTs subjected to sonication treatment for different time intervals. The depicted curves clearly show that the average hydrodynamic diameter of the CNTs progressively decreases with increasing sonication time. Figure 2b reports the average hydrodynamic diameter as a function of sonication time.

Before the sonication, the untreated CNTs show a distribution of hydrodynamic diameters ranging between 100 and 250 nm centered at 150 nm, that is, in the range of the value reported for acid-treated multi walled CNT stable dispersions (150 ± 40 nm) [36]. The CNT lengths, ranging between 0.2 and 1 µm, can be calculated from Equation (1) considering 14 nm as the external radius (from the TEM results) and hydrodynamic diameters reported above.

After 15 min of sonication, the diameter drastically decreases, becoming about half its original size, i.e., 72 nm, and it continues to progressively decrease with the sonication time. Then, for sonication times of 90 and 120 min, the size of the CNTs is no longer altered and the value of the hydrodynamic diameter remains unchanged. It is worth noting that the final value of the hydrodynamic diameter reached at the maximum sonication time is about 33 nm, indicating that the applied sonication treatment has a dramatic effect on the CNTs’ length and, thus, on their aspect ratio. Based on Equation (1), it is possible to estimate that the final length decreases by a factor of seven, considering r unaltered and the hydrodynamic radius before and after 120 min of ultrasonication time.

### 3.2. UHMWPE/CNT Nanocomposites: Rheology, Morphology and Thermo-Oxidation Behaviour

Untreated and sonicated CNTs have been used as nanofillers in UHMWPE-based nanocomposites with the aim of evaluating how the sonication-induced modification of a CNT’s aspect ratio affects the morphology of formulated nanocomposites and their thermo-oxidative resistance.

**Figure 3:**
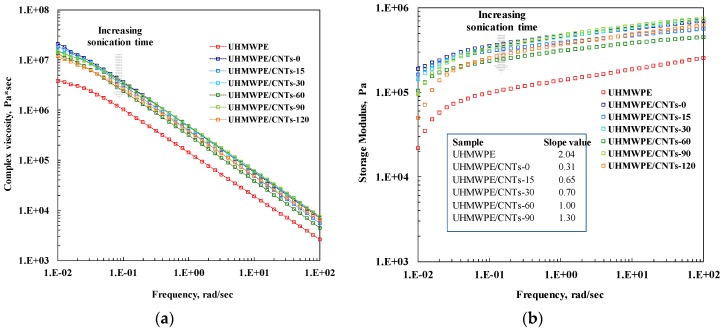
(**a**) Complex viscosity and (**b**) Storage modulus (in the inset the values of the low frequency G′ slopes are reported) as a function of frequency for neat UHMWPE and all CNT-containing nanocomposites.

Rheological analyses are a powerful tool for investigating the microstructure of polymer-based nanocomposites, since the dispersed nanoparticles are able to alter the polymer chain dynamics. Herein, we performed linear viscoelastic measurements to infer the influence of the different aspect ratios of CNTs subjected to sonication treatment on the relaxation processes of UHMWPE macromolecules. In Figure 3a, the trend of complex viscosity as a function of frequency for neat UHMWPE and all CNT-containing nanocomposites is reported. The complex viscosity of the neat polymer matrix exhibits a very short Newtonian plateau, only at the lowest investigated frequencies, followed by a distinct power-law trend for the whole investigated frequency range. Such markedly non-Newtonian behaviour can be attributed to the very high molecular weight of the matrix, whose macromolecules require long relaxation times because of the large content of entanglements. The adding of untreated and sonicated CNTs brings about an increase of the complex viscosity values in the whole tested frequency range, but significant differences emerge when comparing the various nanocomposite samples. Untreated CNTs have a more marked effect on the complex viscosity curve, causing an important increase of the η* values with respect to those of neat matrix and the disappearance of the Newtonian plateau at low frequencies. Nanocomposites containing sonicated CNTs show complex viscosity values higher than those of neat matrix, but the effect of the nanofillers is progressively less pronounced with increasing sonication time. The obtained result can be explained considering that usually a more pronounced increase of the complex viscosity indicates an increasing importance of the tube–tube interactions in dictating the rheological behaviour [37].

In our case, the high aspect ratio of untreated CNTs favors the establishment of intertube interactions and hence adding the nanofillers causes an alteration of the polymer chain dynamics, resulting in rheological behaviour different to that of neat UHMWPE. As the sonication time increases, the CNTs aspect ratio diminishes and the nanofillers are not able to form interconnected structures that modify the macromolecular dynamics. As a result, the effect of adding the nanofillers on the complex viscosity curve is progressively less significant as the sonication time increases, and the η* trend of the nanocomposites becomes similar to that of neat matrix. Similar conclusions can be drawn looking at the trends of storage modulus as a function of frequency reported in Figure 3b. As far as neat UHMWPE is concerned, at low frequencies neat matrix exhibits the typical homopolymer-like terminal behaviour with scaling properties of approximately G′ α ω^2^, see values listed in the inset in Figure 3b. The addition of untreated and sonicated CNTs causes an increase of the G′ values in the whole investigated frequency range; moreover, for all investigated nanocomposites, the terminal behaviour tends to disappear and the dependence of G′ on frequency at low frequencies becomes weaker. However, as already noticed in the analysis of the complex viscosity curves, a significant effect of the CNT sonication time on the nanocomposites’ rheological behaviour can be noticed. For the nanocomposite containing untreated CNTs, the low frequency storage modulus is almost independent of frequency, suggesting a transition from liquid-like to solid-like rheological behaviour for this sample. This non-terminal behaviour can be attributed to the arrangement of CNTs in interconnected structures with the formation of a semi-3D network of nanoparticles that restrains the polymer relaxation process. Nanocomposites containing CNTs treated for low sonication times, i.e., CNT-15 and CNT-30, show similar solid-like rheological behaviour, although the values of the low frequency slope of the storage modulus are higher than that of CNT-0 containing nanocomposite. Otherwise, the rheological behaviour of the nanocomposites containing CNTs treated for long times became progressively similar to that of neat matrix with the increase of the sonication time, indicating that the low aspect ratio of the sonicated CNTs does not allow the CNTs to physically bridge and to form the percolation network.

**Figure 4:**
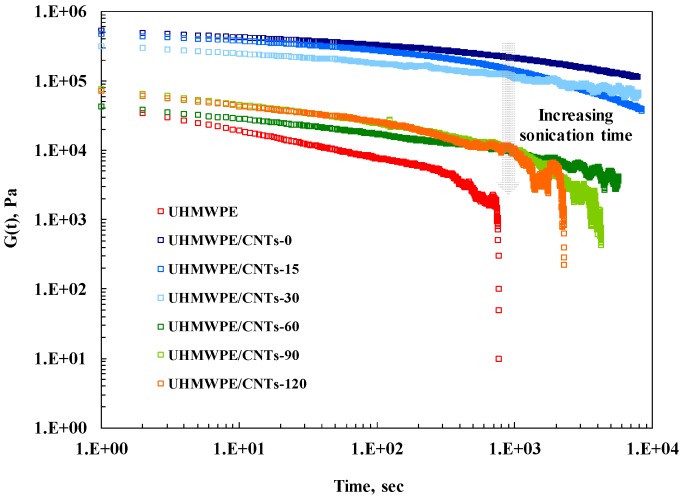
Stress relaxation behaviour of neat UHMWPE and all CNT-containing nanocomposites.

To sum up, as the sonication time increases, the aspect ratio of the CNTs diminishes due to shortening, and this issue implies that the sonicated CNTs are no longer able to hinder the polymer chain relaxation processes. To confirm this feature, stress relaxation measurements were performed and the obtained results are reported in Figure 4, where the relaxation modulus of investigated nanocomposites is compared to that of UHMWPE. The modulus G(t) of CNT-containing nanocomposites is higher than that of neat matrix and, similarly to what was observed through linear viscoelastic measurements, the enhancement of G(t) is more pronounced for untreated CNTs and progressively diminishes with increasing CNT sonication times. Furthermore, while at short times the stress relaxation behaviour is quite similar for nanocomposites and unfilled matrix, at long times different relaxation kinetics can be observed. In particular, neat UHMWPE and nanocomposites containing CNTs sonicated for long times relax like a liquid, indicating that the presence of treated CNT-60, CNT-90 and CNT-120 does not affect the chain relaxation spectrum of UHMWPE. In contrast, adding untreated and CNTs sonicated for short periods significantly modified the polymer chains’ relaxation and the nanocomposites showed a pseudo-solid-like behaviour. Indeed, the stress relaxes to an equilibrium value rather than to zero and the occurrence of this phenomenon can be attributed to the formation of a three-dimensional superstructure of CNTs within UHMWPE.

**Figure 5:**
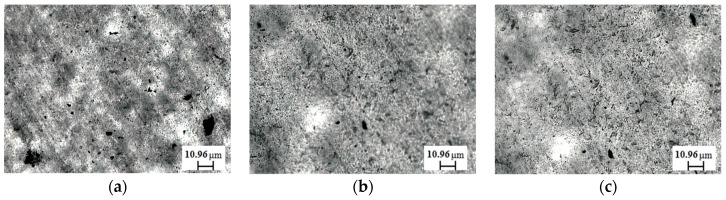
Representative optical micrographs of: (**a**) UHMWPE/CNT-0; (**b**) UHMWPE/CNT-60; (**c**) UHMWPE/CNT-120.

In order to gain insight into the state of dispersion of untreated and sonicated CNTs within polymer matrixes, morphological observations through optical microscopy were carried out and in Figure 5 representative optical micrographs are reported (the embedded CNTs appear as the dark phase). It is evident from the optical observations that the distribution of both untreated and sonicated CNTs is quite uniform and homogeneous, although in the nanocomposite containing untreated CNTs, some agglomerates can be observed. The sonication treatment, thus, is beneficial in enhancing the extent of CNT dispersion. According to the literature, the shortening of CNTs facilitates their dispersion within the polymer matrix due to the decreasing degree of physical interaction between the CNTs themselves. Indeed, as the length of the CNTs diminishes, the contact area between nanofillers becomes lower and decreases the number of physical entanglements between nanotubes [38,39].

**Figure 6:**
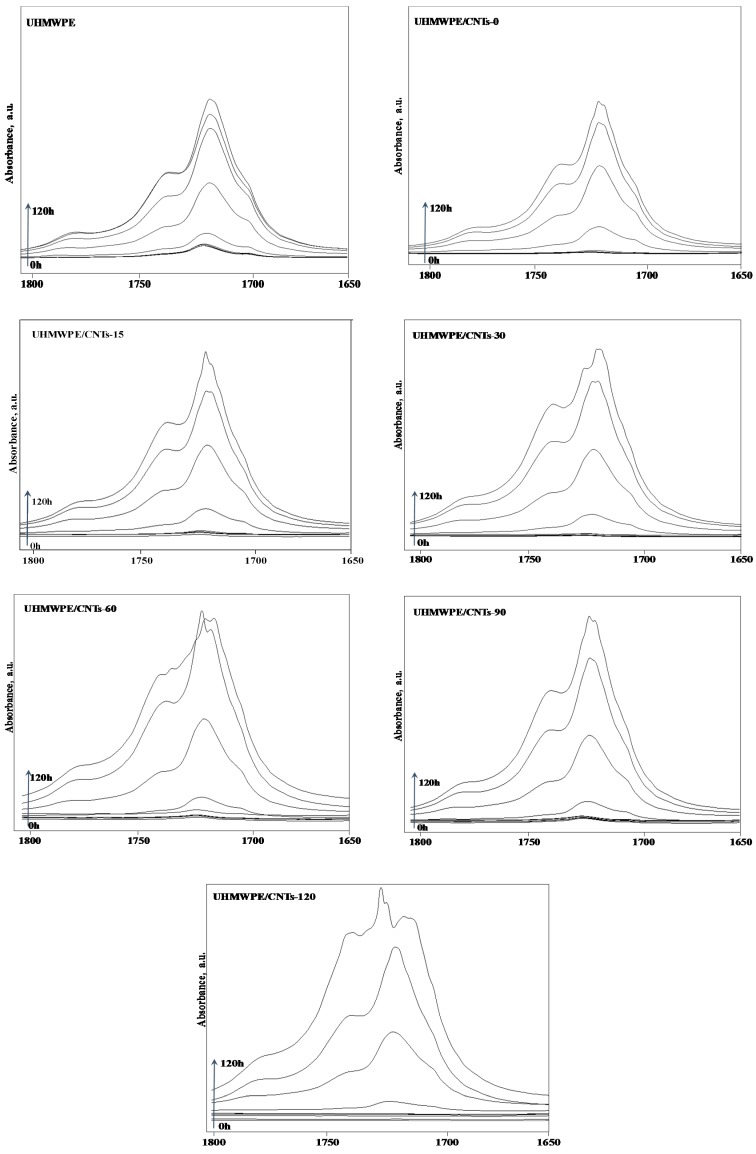
FTIR spectra collected during thermo-oxidative treatment for neat UHMWPE and CNT-containing nanocomposites.

**Figure 7:**
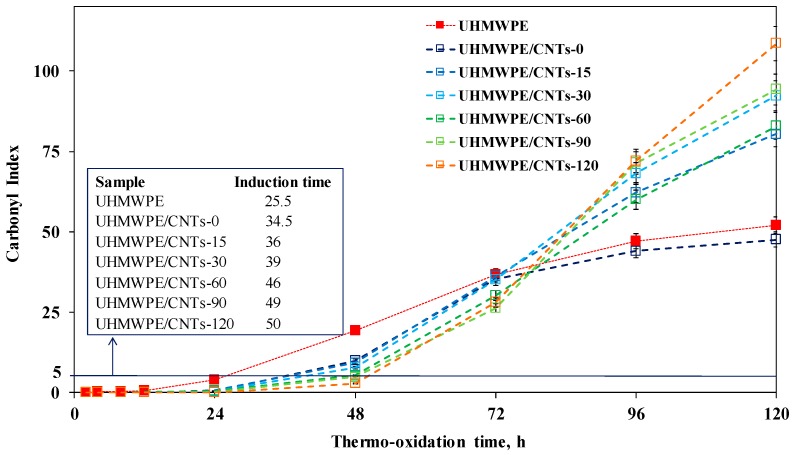
Carbonyl index as a function of thermo-oxidation time for neat UHMWPE and CNT-containing nanocomposites (in the inset the values of the induction time are listed).

The effect of the sonication-induced shortening of CNTs and of the consequent modification of the nanofiller distribution on the thermo-oxidative behaviour of UHMWPE-based nanocomposites was investigated through the analysis of the evolution time of FTIR spectra collected on neat matrix and nanocomposites during an oxidative treatment performed in an air oven at 120 °C. In Figure 6, the FTIR spectra for neat UHMWPE and all CNT-containing nanocomposites are shown. In particular, the progress of the degradative processes was followed through the monitoring of the carbonyl index (CI). The CI refers to the area of the complex peak in the FTIR spectrum in the range 1850–1600 cm^−1^, thus reflecting the formation of carboxylic acids, ketones, esters and lactones, which are the main products of the UHMWPE thermo-oxidation [40,41]. The calculated CI values for neat matrix and all formulated nanocomposites are reported in Figure 7, as a function of the thermo-oxidation time, along with the values of the induction time, defined as the time needed for CI reaches the value of five [42].

Let us now consider the thermo-oxidative behaviour of UHMWPE and nanocomposites at short oxidation times (i.e., until 72 h). Neat UHMWPE shows a sudden increase of carbonyl formation, indicating that the degradation of neat matrix begins in the early stage of the oxidative treatment. The nanocomposite containing untreated CNTs shows improved thermo-oxidative resistance, with an increase of the induction time compared to that of neat matrix, and this result can be ascribed to the well-documented radical scavenging activity of CNTs. Indeed, it is known from the literature that CNTs, because of their peculiar electronic structure, are able to protect polymers against degradative phenomena, acting as antioxidants and interrupting the chain propagation during polymer degradation [43,44]. As far as the nanocomposites containing sonicated CNTs are concerned, the build-up curves of CI reveal a marked influence of the CNT sonication time on the oxidation rate; specifically, as a function of the sonication time, an increase of the stabilizing action of CNTs can be observed. According to the literature, the radical scavenging activity of CNTs can be amplified through the increase in the number of structural defects on the CNT surface [45]. The last can be explained considering that the antioxidant feature of CNTs is attributed to the presence of acceptor-like localized states, which results from lattice defects on CNTs. As probed through Raman analysis, the content of structural defects progressively increases with the sonication time; therefore, the amplified antioxidant action of sonicated CNTs can be attributed to their higher amount of lattice defects compared to untreated nanotubes. After 72 h of thermo-oxidative treatment, an unexpected inversion of the CI build-up curves occurs. The slope of the CI curves for neat UHMWPE and nanocomposite-containing CNT-0 decreases, indicating a reduction of the rate of formation of oxidized species. In contrast, the nanocomposites containing sonicated CNTs exhibit a faster degradation kinetic and the growth of the CI values increases as a function of the CNT sonication time. The obtained results could be explained considering that, as reported in the literature, the stabilizing action of CNTs can be compromised if local increases of the temperature at the polymer/nanoparticle interface occur [46]. In our case, the oxidation treatment is carried-out at high temperature (i.e., 120 °C); therefore, after a certain period of exposure, nanocomposites are no longer able to dissipate the thermal energy and the local temperature starts to increase, causing in turn an acceleration of degradative phenomena.

The worsening of the thermo-oxidative resistance of the nanocomposites as a function of the CNT sonication time could be ascribed to the lower thermal conductivity of shorter CNTs with respect to that of long nanotubes. Indeed, it is well documented in the literature that CNTs with higher aspect ratios can provide a larger enhancement of nanocomposite thermal conductivity than CNTs with a lower aspect ratio [19,47]. The last because long CNTs are able to form a percolation network within the polymer matrix in a more efficient way than short CNTs, as already demonstrated through rheological analyses.

## 4. Conclusions

Nanocomposites based on UHMWPE and CNTs subjected to a sonication treatment for different time intervals were produced with the aim of investigating the influence of the sonication-induced shortening of the CNTs on their rheological behaviour and thermo-oxidative stability. The effective reduction of the length of the original CNTs was probed through accurate spectroscopic and morphological analyses. The study of the nanocomposites’ rheological behaviour revealed that the sonication treatment strongly affected the rheological behaviour of the CNT-containing nanocomposites, since the reduction of the nanofiller aspect ratio counteracts the formation of a percolation network within a polymer matrix; as a result, the rheological behaviour of nanocomposites containing CNTs sonicated for long times is quite similar to that of neat matrix. The reduction of the aspect ratio in sonicated CNTs also dramatically affects the thermo-oxidative resistance of UHMWPE-based nanocomposites and results in a progressive deterioration of the long-term stability of nanocomposites with increasing sonication times. The obtained results can be explained invoking the low thermal conductivity of nanocomposites containing short CNTs, that do not allows the dissipation of accumulated thermal energy, with a consequent increase of the local temperature and, thus, of the oxidation kinetics.
